# Overexpression of Hydroxynitrile Lyase in Cassava Roots Elevates Protein and Free Amino Acids while Reducing Residual Cyanogen Levels

**DOI:** 10.1371/journal.pone.0021996

**Published:** 2011-07-25

**Authors:** Narayanan N. Narayanan, Uzoma Ihemere, Claire Ellery, Richard T. Sayre

**Affiliations:** Donald Danforth Plant Science Center, St. Louis, Missouri, United States of America; Instituto de Biología Molecular y Celular de Plantas, Spain

## Abstract

Cassava is the major source of calories for more than 250 million Sub-Saharan Africans, however, it has the lowest protein-to-energy ratio of any major staple food crop in the world. A cassava-based diet provides less than 30% of the minimum daily requirement for protein. Moreover, both leaves and roots contain potentially toxic levels of cyanogenic glucosides. The major cyanogen in cassava is linamarin which is stored in the vacuole. Upon tissue disruption linamarin is deglycosylated by the apolplastic enzyme, linamarase, producing acetone cyanohydrin. Acetone cyanohydrin can spontaneously decompose at pHs >5.0 or temperatures >35°C, or is enzymatically broken down by hydroxynitrile lyase (HNL) to produce acetone and free cyanide which is then volatilized. Unlike leaves, cassava roots have little HNL activity. The lack of HNL activity in roots is associated with the accumulation of potentially toxic levels of acetone cyanohydrin in poorly processed roots. We hypothesized that the over-expression of HNL in cassava roots under the control of a root-specific, patatin promoter would not only accelerate cyanogenesis during food processing, resulting in a safer food product, but lead to increased root protein levels since HNL is sequestered in the cell wall. Transgenic lines expressing a patatin-driven HNL gene construct exhibited a 2–20 fold increase in relative HNL mRNA levels in roots when compared with wild type resulting in a threefold increase in total root protein in 7 month old plants. After food processing, HNL overexpressing lines had substantially reduced acetone cyanohydrin and cyanide levels in roots relative to wild-type roots. Furthermore, steady state linamarin levels in intact tissues were reduced by 80% in transgenic cassava roots. These results suggest that enhanced linamarin metabolism contributed to the elevated root protein levels.

## Introduction

Cassava (*Mannihot esculenta*) has shaped the cultures, diets and economies of more than 800 million people worldwide particularly in Sub-Saharan Africa. Cassava has many desirable traits for a staple crop including its ability to grow in poor soils, drought resistance, and ability to be harvested as soon as six months and as late as 3 years after planting [1]. Its wide harvest window enhances food security and offers increased harvesting flexibility for resource-poor farmers [2]. Although cassava is rich in carbohydrates (30% starch per gram fresh weight), it is a poor source of protein as well as many essential micronutrients and vitamins [3]. Cassava has the lowest protein-to-energy ratio of any major staple food crop in the world [4]. A cassava-based diet provides less than 30% of the minimum daily requirement for protein for a typical sized meal (500 gdw). Therefore, additional food sources are required to ensure a balanced diet [5].

Cassava also contains potentially toxic levels of cyanogenic glucosides including linamarin (95%) and lotaustralin (5%) [6]–[8]. Leaves have high cyanogenic glucoside levels (5 g linamarin/kg fresh weight), whereas roots have 10–20 fold lower levels [9].Various health disorders have been associated with the consumption of poorly processed cassava due to the presence of residual cyanogens (linamarin and acetone cyanohydrin). Chronic, low-level cyanide exposure from consumption of poorly processed cassava is associated with the development of goiter and tropical ataxic neuropathy, a nerve-damaging disorder that renders a person unsteady and uncoordinated [10]. Severe acute cyanide poisoning associated with eating unprocessed cassava, typically during famines, is associated with outbreaks of a debilitating, irreversible paralytic disorder called Konzo. In some cases, consuming unprocessed high cyanogen cultivars results in death [11]. Starving people experiencing protein deficiency are particularly susceptible to cyanide poisoning, as they lack sufficient cysteine in their diet to detoxify cyanide via rhodanese [12]. On the other hand, the cyanogenic glycosides present in intact plant tissues have been shown to have beneficial effects including protection of the plant from herbivory by animals as well as theft deterrence during famines [13], [1].

The physiology and the biochemistry of cyanogensis in cassava have been well studied [7], [14]. Cyanogensis is initiated by rupturing the vacuole which releases linamarin followed by enzymatic deglycosylation of linamarin by the apoplastic enzyme linamarase yielding acetone cyanohydrin [15], [16], [7], [17], [14]. The production of cyanide from acetone cyanohydrin is catalyzed either enzymatically by hydroxynitrile lyase (HNL) or occurs spontaneously at pHs >5.0 or temperatures >35°C [18], [9], [14]. Several attempts have been made to reduce cyanogens in cassava through various biotechnological approaches. Expression of antisense CYP79D1/CYP79D2 (genes encoding cytochrome P450 s) constructs under the control of the leaf-specific Cab1 promoter, resulted in substantial cyanogen reductions in leaves but also in roots [8]. Root linamarin levels in transgenic plants in which the expression of the CYP79D1/CYP79D2 genes was suppressed in leaves were on average only 1% of wild-type levels. Reduction in leaf linamarin synthesis resulted in stunted plants with smaller roots. This phenotype could be complemented, however, by adding reduced nitrogen to the growth media [14]. These and other results indicated that cyanide or nitrile groups derived from linamarin turnover was a substrate for amino acid synthesis in roots [14].

Cassava HNL is a homodimeric protein localized in the apoplast of leaves similar to linamarase [18]. HNLs are also highly conserved among a variety of plants. It has 77% sequence identity with the deduced amino acid sequence of the rubber tree (Figure S1). Significantly, total leaf HNL enzyme activity (apparent V_MAX_) and its affinity (K_m_) for its substrate, acetone cyanohydrin, are similar to those of linamarase for linamarin indicating that HNL activity does not limit cyanogenesis in cassava leaves [9]. In contrast to leaves, however, cassava roots have virtually no HNL activity. Analysis of HNL transcript levels indicated they were <6% of those in leaves [9]. Interestingly, HNL is expressed only in the stems and leaves of sorghum and linen and not in roots [19]. The lack of HNL in roots of cyanogenic plants presumably reduces cyanogenesis in damaged root tissues. The lack of HNL in cassava roots, however, extends the processing time that is required to detoxify cassava foods.

Until recently, it was generally accepted that residual linamarin was the only potential source of cyanide in poorly processed cassava foods since acetone cyanohydrin is chemically unstable and was thought to decompose spontaneously to produce cyanide. The cyanide is then lost from processed cassava foods by volatilization [11]. Free cyanide, however, is not found in cassava foods due to its volatility, thus only residual cyanogens in poorly processed foods are a health concern. In the early 1990s, it was demonstrated that the major residual cyanogen in poorly processed cassava roots was acetone cyanohydrin, and not linamarin [11]. Later, it was demonstrated that HNL was not expressed in roots presumably accounting for the high residual acetone cyanohydrin levels in poorly processed roots [9]. In 2004, transgenic plants expressing HNL in all tissues under the control of the CaMV 35S promoter were shown to have accelerated rates of acetone cyanohydrin conversion to cyanide facilitating the detoxification of cassava foods [20].

Besides their potential toxicity it is now recognized that cyanogenic glycosides play an important role in primary nitrogen metabolism in cassava. It is generally accepted that linamarin is a major transportable form of reduced nitrogen from leaves to roots where it is stored or used for amino acid synthesis [14], [21], [22]. Linamarin is stored in the vacuole or presumably deglycosylated by a generalized β-glucosidase. Acetone cyanohydrin, the product of linamarin deglycosylation, is then either directly assimilated or spontaneously degrades in the cytoplasm to produce cyanide which is then assimilated via β-cyanoalanine synthase to produce β-cyanoalanine and sulfide [23]. Following hydration of β-cyanoalanine to form asparagine, deamination of this amino acid generates aspartate and free ammonia which can be reassimilated by the glutamine synthetase/synthase cycle and used for the synthesis of a diversity of amino acids [24]. Consistent with this hypothesis the activities of β-cyanoalanine synthase and β-cyanoalanine hydrase are 3-fold higher in roots than in leaves [25], [26]. Recent evidence indicates that linamarin metabolism can be accelerated to support protein accumulation in roots [20], [27]. Over-expression of an artificial storage protein in cassava roots not only elevated total root protein levels but also reduced steady state pool sizes of linamarin.

In this work, we show that the root-specific expression of cassava HNL not only increases total root protein levels three fold approaching the target values for a nutritionally balanced meal but accelerates cyanogenesis during food processing resulting in a safer and more nutritious food product. We demonstrate that the over-expression of HNL driven by the patatin (root-specific) promoter results in a 2–20 fold increase in relative mRNA expression in roots when compared with wild type, and a 5–6 fold increase in expression when compared with CaMV 35S HNL transgenic lines. We also demonstrate that after just 90 minutes processing time, the roots of transgenic lines had substantially lower cyanide and acetone cyanohydrin levels than wild-type lines. The transgenic HNL lines also exhibited reduced (53–74%) root linamarin steady state levels. Since HNL is made up of 50% essential amino acids and is consumed by humans when leaves are eaten, we propose that over-expression of HNL in roots provides a safe and effective way of elevating root protein nutrition while also reducing cyanogen toxicity.

## Results

### Molecular characterization of cassava HNL transgenic plants

**Figure 1:**
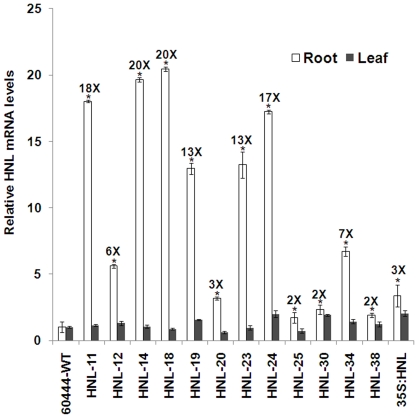
Overexpression of HNL in cassava roots. Relative expression levels (Q-RT-PCR) of HNL in twelve independent patatin promoter and 35S promoter HNL transgenic line. Tissues (both roots and leaves) were collected at 1.5 month old *in vitro* stage. HNL expression was compared and normalized to tubulin. Wild type expression values were adjusted to a value of 1 and all other expression values were normalized relative to this tissue. The number above the white bars (roots) indicates the fold increase compared to wild type. Error bars represent SE of three biological replicates. The asterisk (*) indicates statistically significant differences between wild-type and transgenics, determined by Student's t-test, with P<0.05.

Using a high-throughput friable embryogenic callus (FEC's) transformation system, forty-two independent putative (antibiotic resistant) transgenic lines were obtained with 33 lines shown to be positive for the presence of the HNL transgene by PCR analysis (Figure S2). Previous analyses had indicated that transgenics containing single transgene copy numbers typically had the highest levels of transgene expression. In order to determine the HNL gene copy number in transgenic lines, the 35S promoter was targeted for dot blot analysis and quantification. Seven lines were shown to have a single transgene copy, while sixteen lines had two transgene copies, and ten lines had three copies of the transgene (Figure S2A). To verify expression of the transgene, semi-quantitative PCR was performed in leaves and roots of transgenic HNL and wild-type lines. HNL expression was normalized to tubulin and compared to expression levels in wild-type lines. Transgenic lines had HNL expression levels ranging from 2- to 20-fold greater than wild-type plants and ranging from 5- to 6-fold greater than the CaMV 35S HNL transgenic lines (Figure 1). Among the twelve independent transgenic lines analyzed, HNL-11, HNL-14, HNL-18 and HNL-24 exhibited the highest HNL expression relative to wild type (Figure 1). There was no significant difference in HNL mRNA expression in the leaves of patatin-driven HNL transgenics compared to wild-type plants (Figure 1). In contrast, CaMV 35S: HNL transgenic lines had elevated HNL expression in the leaves relative to patatin:HNL transgenic lines and wild type (Figure 1).

### Specific activity of hydroxynitrile lyase increases in transgenic roots

**Figure 2:**
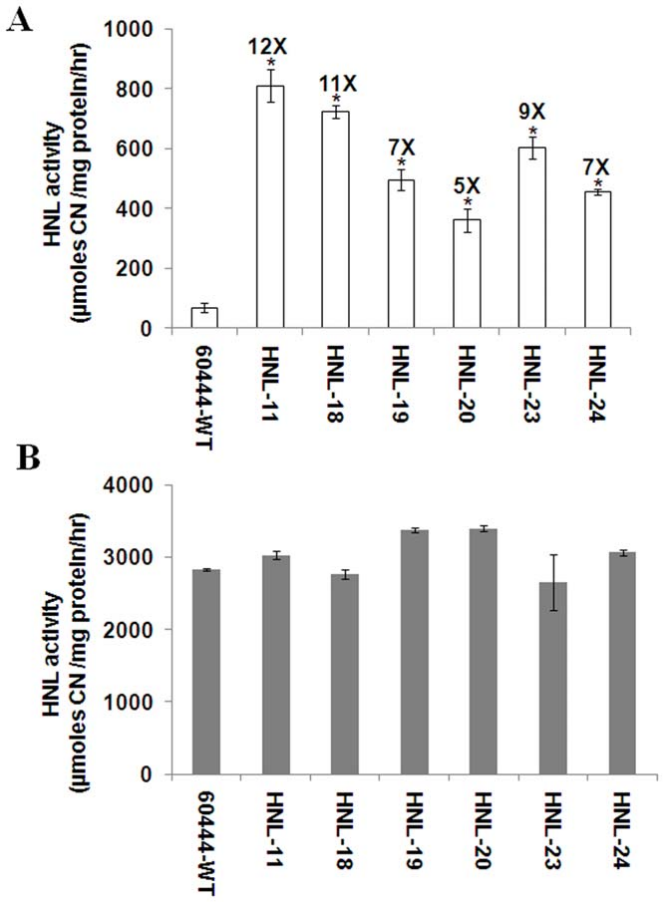
HNL activity increases in transgenic roots. Relative HNL activity of (A) roots; (B) leaves. Protein extracts were obtained from root and leaf tissues and HNL enzyme activity was measured colorimetrically. Data are presented as relative amounts of cyanide per mg of protein per hr. Error bars indicate SE of the mean of three biological replicates. The asterisk (*) indicates statistically significant differences between wild-type and transgenics, determined by Student's t-test, with P<0.05.

HNL enzyme activity was measured in both roots and leaves of transgenic and wild-type cassava lines. Analysis of the HNL activity in roots indicated that there was as much as a 12-fold increase in enzyme activity relative to wild type (Figure 2A). Transformed cassava lines (HNL-11, HNL-18, HNL-19, HNL-20, HNL-23, and HNL-24) had HNL activities ranging from 811to 455 µmoles HCN/mg protein/h, while wild-type plants had HNL activity rates of 69 µmoles HCN/mg protein/h. Consistent with the mRNA expression profiles, no significant differences in HNL activity were observed in leaves of transgenic and wild-type plants (Figure 2B).

### Western blot analysis

**Figure 3:**
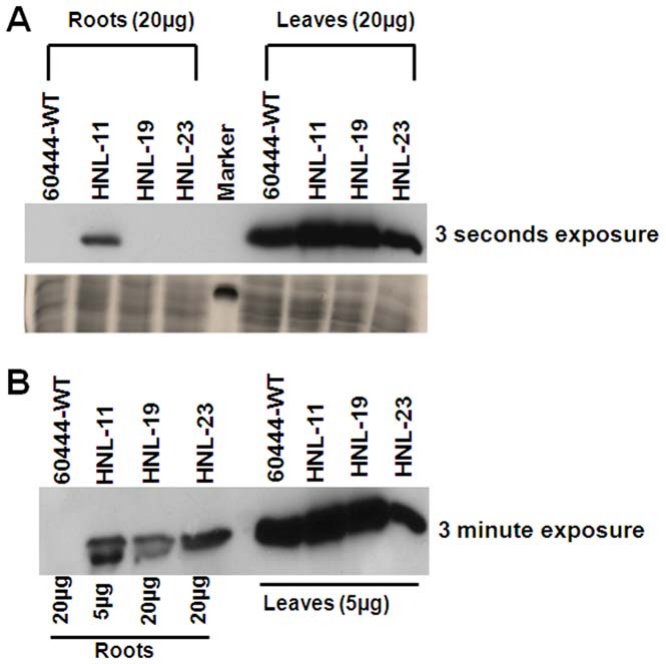
Immunoblots of transgenic cassava expressing HNL protein. Different amounts of protein (as indicated) from transgenic and control roots and leaves were separated by SDS-PAGE and transferred to nylon membranes. Membrane was probed first with an anti-HNL antibody (1∶1000) followed by secondary antibody (Anti-rabbit IgG antibody conjugated to horse-radish peroxidase) at 1∶10000 dilution. Detection was performed by chemiluminescence (Sigma) according to manufacturer's instructions followed by exposure to X-ray films for (A) 3 seconds and (B) 3 minutes.

To compare post-translational expression levels of HNL in leaves and roots of transgenic and wild-type plants, HNL protein abundance was evaluated by western blot analysis. Within 3 seconds exposure to the immuno-detection system both wild-type and transgenic leaf samples exhibited a distinct 29 kDa protein band corresponding to the molecular weight of HNL (Figure 3A). Under similar detection conditions none of the root samples exhibited an immuno-labeled HNL protein with the exception of HNL-11. HNL-11 root protein extracts exhibited a clear immuno-detectable band within 3 sec exposure indicative of a high level of HNL accumulation (Figure 3A). These results were consistent with the high HNL enzyme activity observed in this transgenic line. In order to detect lower HNL levels in some transgenics western blots were exposed for longer times (Figure 3B). Within 3 minutes exposure, detectable HNL was also observed from root protein extracts of transgenic lines HNL-19 and HNL-23, but not with wild type root extracts (Figure 3B).

### Over expression of HNL reduces cyanide levels in transgenic roots

**Figure 4:**
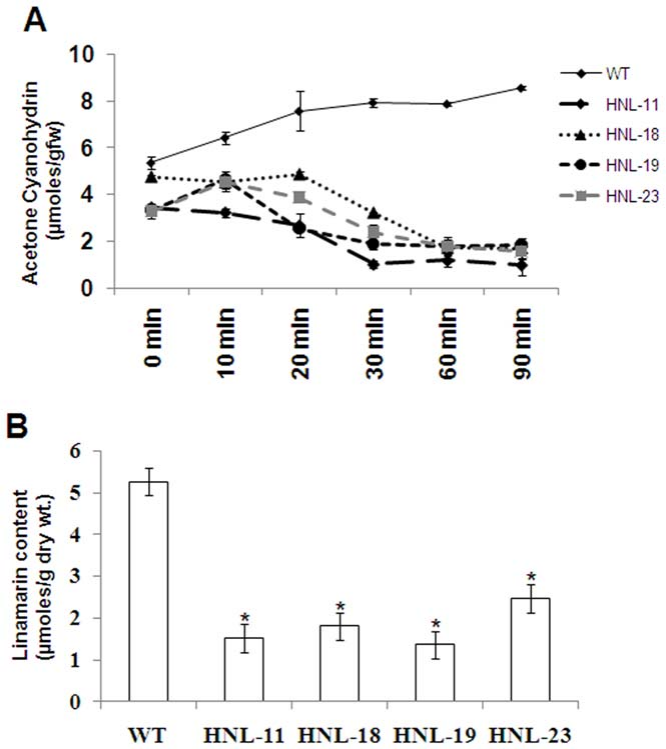
Overexpression of HNL decreases cyanide levels in transgenic roots. (A). Acetone cyanohydrin levels were measured using seven month old root cortex tissues assayed at different time intervals post-homogenization. Data are presented as the amount of acetone cyanohydrin levels calculated as the difference between the two assays {(acetone cyanohydrin + cyanide assay) – (cyanide assay)}. Error bars indicate SE of the mean of three biological replicates. (B). GC-MS quantification of root linamarin contents in wild-type and transgenic plants. Samples were normalized with internal standard phenyl β-glucopyranoside (PGP). Linamarin content is expressed as µmoles per gram dry weight. Error bars indicate SE of the mean of four biological replicates. The asterisk (*) indicates statistically significant differences between wild-type and transgenics, determined by Student's t-test, with P<0.05.

To determine whether the overexpression of HNL enhanced root cyanogensis, we measured linamarin-derived acetone cyanohydrin turnover and cyanide production in roots (homogenized at pH 5.0 to prevent spontaneous acetone cyanohydrin decomposition) at various time points after homogenization. In this assay system any cyanide generated is the result of enzymatic production from acetone cyanohydrin [20]. As shown in Figure 4A, roots from transformed plants having elevated HNL levels had an 80-90% reduction in total acetone cyanohydrin levels relative to wild type within 90 minutes following tissue disruption. Previously, we had shown that incubation of homogenized roots for up to 2 hours was required for complete conversion of linamarin to acetone cyanohydrin in CaMV 35S HNL transgenic lines [20]. Specifically, transgenic line HNL-11 (highest expressor) had nearly a 10-fold reduction in cyanogens (0.99 µmoles/g fresh weight) relative to control plants (8.56 µmoles/g fresh weight) 90 min post-homogenization (Figure 4A). The three additional transgenic lines screened (HNL-18, HNL-19 and HNL-23) also had substantially lower residual acetone cyanohydrin levels (1.7, 1.83 and 1.57 µmoles/g fresh weight respectively, Figure 4A) relative to wild-type plants.

### Impact of HNL overexpression on steady state linamarin levels

To determine the effect of over expression of HNL on nitrogen metabolism and linamarin pool sizes the linamarin content in both leaves and roots was quantified by GC-MS. Significantly, GC-MS analysis of linamarin levels does not depend on the complete linamarase dependent conversion of linamarin to acetone cyanohydrin and cyanide as is required by the indirect CN-electrode-based determination of linamarin content [27]. Furthermore, inadvertent linamarin losses occurring during sample processing are normalized by the inclusion of an exogenously added internal standard (phenyl β-glucopyranoside), in the GC-MS linamarin quantification method used in this study [8]. Adding confidence to the accuracy of the linamarin analyses by GC-MS, we note that cyanogen quantification by the indirect colorometric quantification of CN gave results essentially identical to those obtained by GC-MS (Figure 4A and 4B). Significantly, the average steady-state linamarin content (ranging from 25–30 µmoles/g dry weight) of leaves from patatin: HNL transgenic lines was nearly identical to that of wild-type plants (Figure S3) indicating that overexpression of HNL in roots had no affect on leaf linamarin pool sizes. Importantly, the linamarin content of wild-type (cultivar TMS-60444) leaves was similar to those previously reported for other cassava cultivars [reviewed in 7, 15]. In contrast, the leaf linamarin levels reported for the identical cultivar (TMS-60444) using the cyanide-electrode assay system averaged 3.7 µmole/gdw (100 ppm) or 10 fold lower than the measurements made by GC-MS [27]. Finally, in contrast to the HNL overexpressing plants, transgenic plants overexpressing zeolin in storage roots had an apparent 50% reduction in reported leaf linamarin levels [27]. The effect of an apparent 50% reduction in leaf cyanogen levels on herbivore susceptibility remains to be determined.

In contrast to leaves, the average steady-state linamarin content of roots overexpressing HNL was 53–74% lower in transgenic lines than in wild-type plants (Figure 4B). Transgenic lines HNL-19, HNL-11, HNL-18 and HNL-23 exhibited reductions in linamarin content ranging from 53–74% (1.36, 1.52, 1.8 and 2.47 µmoles/g dry weight respectively) relative to wild-type plants (5.26 µmoles/g dry weight) (Figure 4B). These results are consistent with previous observations on linamarin content of cassava roots but are nearly100-fold greater than the values (52 nmole/gdw; 1.4 ppm) reported for roots of the same cassava cultivar using the cyanide-electrode assay system [7], [15], [27]. Overall, these results support the hypothesis that linamarin may be utilized for amino acid and protein synthesis in roots [8]. The impact of these lianamrin reductions on root susceptibility to herbivory also remains to be determined.

### Over expression of HNL increases free amino acid and total protein levels

**Figure 5:**
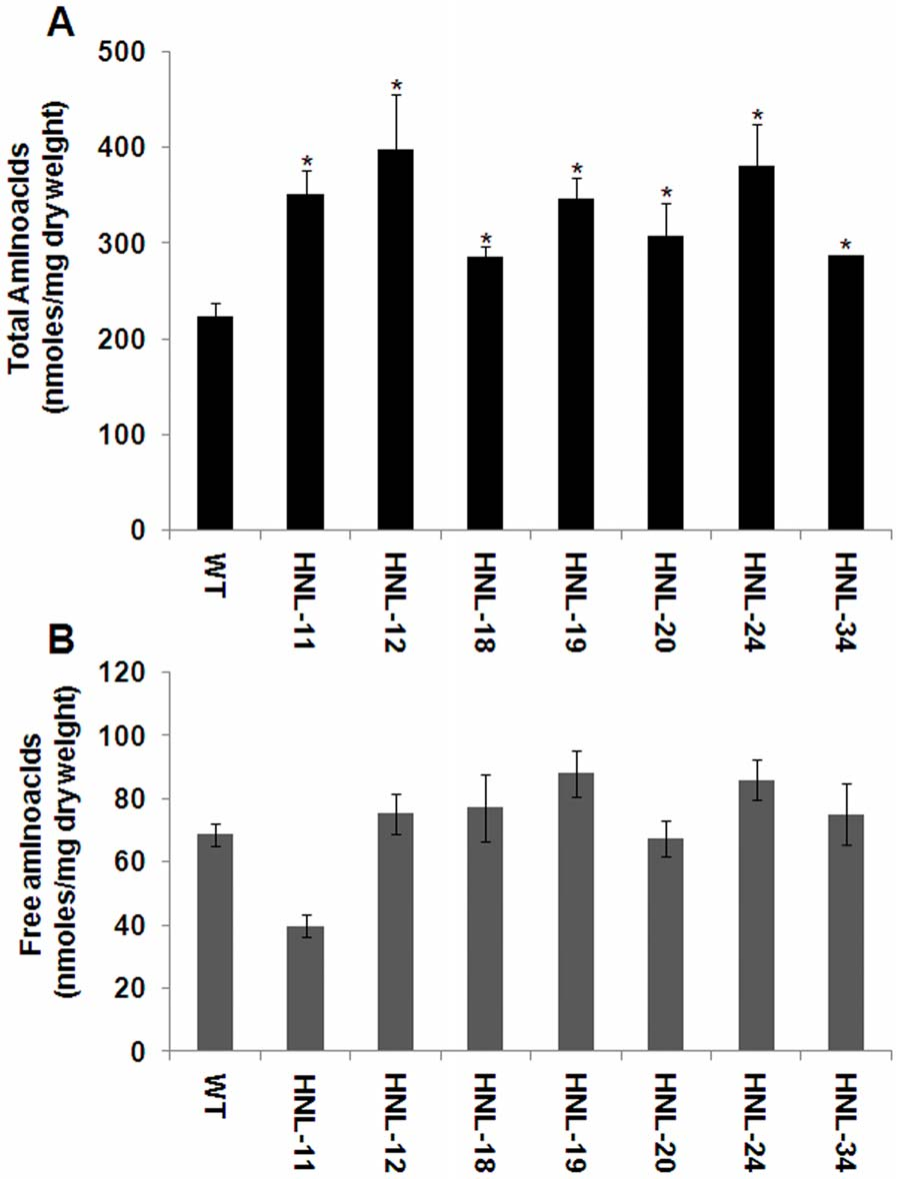
Total and free amino acid content of transgenic and wild-type cassava roots. (A) Measurement of total protein hydrolyzed and free amino acid concentrations in transgenic and wild-type roots. Samples were hydrolyzed with HCl and subjected to ACQUITY UPLC® System. Data are presented as nmoles/mg total dry weight. Error bars indicate SE of the mean of two biological replicates. The asterisk (*) indicates statistically significant differences between wild-type and transgenics, determined by Student's t-test, with P<0.05. (B) Measurement of free amino acid concentrations of transgenic and wild-type roots. Data are presented as nmoles/mg total dry weight of tissue. Error bars indicate SE of the mean of two biological replicates.

It has been proposed that cassava plants use linamarin as a transportable source of reduced nitrogen for amino acid synthesis in roots [8]. In order to study whether overexpression of HNL altered root amino acid levels available for protein synthesis, free and total (free plus protein) amino acid levels in roots of seven month transgenic and wild-type plants were analyzed. The levels of total free amino acids of the different transgenic lines are shown in Figure 5A and individual amino acid compositions are shown in Figure S4. Transgenic lines exhibited a 1.5–2 fold increase in root total amino acids consistent with the increase in total root protein. Relative to wild type the largest increases in total amino acids were observed for the amino acids Gly, Asp, Glu, and Arg in transgenic plants (Figure S4). Other amino acids such as His, Ser, Thr, Ala, Pro, Lys, Val, Ileu and Leu also showed increased levels in transgenics compared with wild type (Figure S4). Relative to wild type, however, only HNL-11 had a significant reduction in free (non-protein) amino acid levels (Figures 5B and S5). Those free amino acids exhibiting the greatest reductions were Asn, Ser, Arg, Asp, Glu, Pro, Lys, and Ileu (Figure S5). Many of these amino acids (Arg, Asn, Asp, and Glu) are involved in reduced nitrogen cycling and generalized amino acid synthesis (Figure S6).

**Figure 6:**
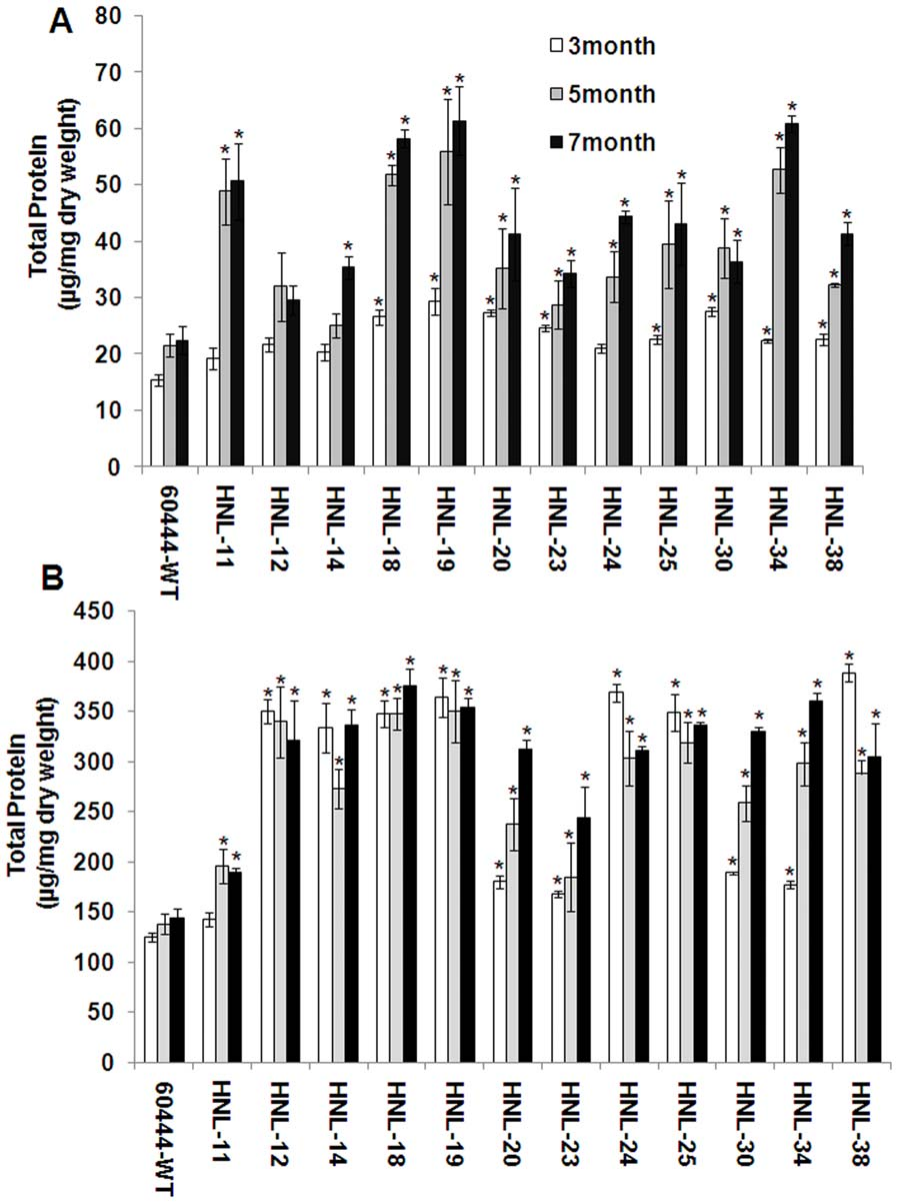
Overexpression of HNL increases root protein concentrations. Measurement of total protein concentrations of (A) roots, and (B) leaves of transgenic and control plants. Total protein was extracted and measured using CB-X assay kit. Data are presented as total protein in µg/mg dry weight. Error bars indicate SE of the mean of three biological replicates. The asterisk (*) indicates statistically significant differences between wild-type and transgenics, determined by Student's t-test, with P<0.05.

Significantly, the HNL transgenics had 2 to 3-fold greater soluble root protein levels in seven month old roots than wild-type plants (Figure 6A). Furthermore, root protein levels increased with age indicating the transgenics were accumulating protein over time. The HNL transgenic had a 2–3 fold increase in root protein from months 3–7 after planting compared to only a 45% increase in wild type. This increase in root protein levels corresponds with the increase in starch accumulation and potential carbohydrate induction of the root-specific patatin promoter (Figure 6A). Similarly, most of the transgenic lines also had higher leaf protein concentrations (2–3 fold) relative to wild-type plants consistent with the hypothesis that leaves are a major nitrogen (protein) sink in cassava and a potentially stronger sink than proteins targeted for overexpression in roots (Figure 6B). Significantly, the total root protein content measured colorometrically corresponded well with the total free and protein amino acid levels determined by UPLC (see Methods).

## Discussion

**Figure 7:**
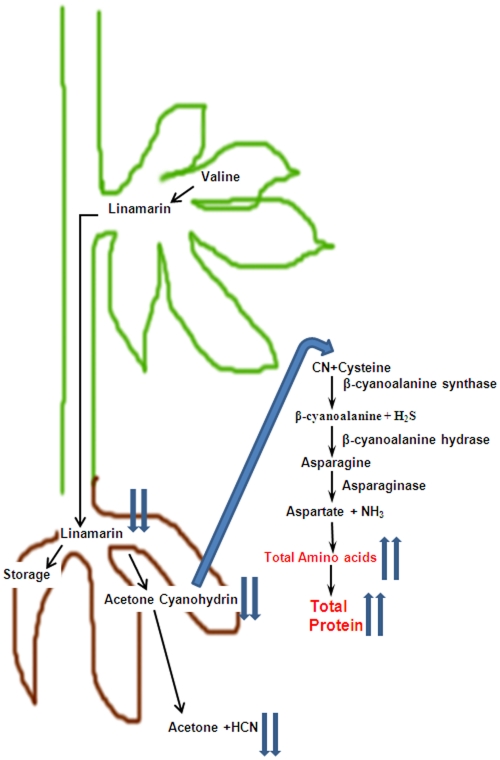
Hypothetical model of cyanogen metabolism in cassava roots. Proposed pathway of linamarin synthesis and breakdown in cassava roots. Transgenic cassava overexpressing HNL leads to decrease in steady state levels of linamarin in roots and reductions in both acetone cyanohydrin and cyanide levels after processing. In addition, there is an increase in total HNL protein which ultimately leads to increase in total protein level in roots. Two small blue arrows indicate either decrease or increase levels in roots.

As many as 12,000 plant species including many important crops such as cassava, sorghum, almonds, lima beans and white clover contain cyanogenic glucosides. Cyanogenic glycosides are nitrile containing plant secondary compounds generally synthesized from amino acids that yield cyanide upon enzymatic breakdown [28]–[31]. The major (95% of the total) cyanogenic glucoside in cassava is linamarin, derived from valine [7]. Several transgenic approaches have been attempted to reduce or eliminate cyanogens in cassava foods [8], [20], [22]. Leaf-specific inhibition of CYP79D1/D2 expression lead to a 99% reduction in root cyanogen levels indicating that the linamarin present in roots was largely synthesized in leaves and transported to roots [14]. It is also well established that elevated levels of HNL in cassava roots accelerate the conversion of acetone cyanohydrin to cyanide facilitating the detoxification of cassava roots by cyanide volatilization during food processing [20]. Here, we demonstrate that the specific overexpression of HNL in roots not only accelerates root cyanogenesis resulting in safer food products, but increases root protein concentrations by three fold (Figure 7).

We show that root-specific expression of HNL resulted in a 5–6 fold increase in relative HNL mRNA abundance in roots relative to HNL transcript levels observed in transgenic plants in which HNL expression was driven by the CaMV 35S promoter (Figure 1). Similar results were obtained when *CrtB* (genes encoding phytoene synthase) was overexpressed in potato using the tuber-specific patatin promoter [32]. In this work, the increase in total root protein levels in HNL transgenic plants between 3 and 7 months after planting suggests the effect of root-specific patatin promoter is cumulative and that the targeting of HNL to the apopolast allows for continuous protein accumulation over time (Figure 6A). Transgenic plants had a 5–12-fold increase in root HNL enzyme activity compared with wild-type plants (Figure 2A). Previously, an 8 to 13 fold increase in root HNL activity in transformed plants relative to wild type was observed when using the CaMV 35S promoter to drive HNL expression in roots as well as leaves [20]. These results suggest that root HNL levels are not solely determined by the type of gene promoter used to drive its expression but may be limited by the capacity of roots to accumulate proteins due in part to the strong nitrogen sink strength of leaves. Regardless of the HNL gene promoter used, root HNL activities in transgenic plants were substantially less than those of leaves [9]. Significantly, many African societies consume cassava leaves following extensive preparation to remove cyanogens. As a result, HNL is regularly consumed by these groups. At present, there are no known allergies or identifiable allergenic determinants in cassava HNL. Thus, over-expression of HNL in cassava roots should not present a potential health risk. In contrast, it has been noted that zeolin has a potential allergenic domain, corresponding to the phaseolin β-conglycinin domain [27], [33]. Thus, it is unlikely that zeolin expressing cassava plants will be acceptable for human consumption.

To establish whether the observed increase in root HNL activity was correlated with greater HNL protein abundance, HNL immuno-blots were carried out for transgenic and wild-type root and leaf tissues. It was demonstrated earlier that HNL was not detectable in wild-type cassava roots by western blot analysis using polyclonal antibodies generated in mice [9]. In our study, transgenic line HNL-11, which had the highest HNL enzymatic activity, had the greatest immuno-detectable HNL levels in roots (Figure 3A) indicative of a strong correlation between HNL enzyme activity and protein abundance in transgenic roots. Interestingly, when root HNL levels were compared to total leaf protein levels the transgenic line HNL-19, which had one of the lowest root HNL levels (activity and immuno-detectable), had the highest total leaf protein level. These results suggest that other proteins may be accumulating in storage roots or in leaves of transgenic plants over-expressing HNL, perhaps related to the accelerated turnover of linamarin in roots of transgenic plants.

Generating a strong protein sink in cassava roots would be expected to alter root linamarin metabolism. Cyanogenic glucosides have previously been shown to serve as sources of reduced nitrogen which can be assimilated by the β-cyanoalanine synthase pathway for the production of asparagine, aspartate and free ammonia and in turn free amino acids [33]. Cyanogenic glycoside catabolism for protein synthesis was initially observed in germinating rubber seedlings [34]. Rubber tree seed endosperm contains high concentrations of linamarin. During germination and rubber tree plantlet development 85% of the seed linamarin reserves are metabolized with negligible amounts of gaseous HCN liberated. Due to the high levels of the cyanide assimilating enzyme β-cyanoalanine synthase in young rubber tree seedling tissues, it was proposed that linamarin transported from the seed endosperm to the growing seedling served as a source of reduced nitrogen for seedling metabolism. In cassava, it has also been proposed that linamarin is used as a transportable source of reduced nitrogen for amino acid synthesis in roots [8], [14]. The conversion of cyanide to asparagine in cassava has been well demonstrated using radiolabeled precursors [35]. In our work, the average root linamarin content of transgenic lines was 53–74% lower than linamarin levels in wild-type plants consistent with its utilization for protein synthesis and accumulation in HNL overexpressing roots (Figure 4B). Consistent with the metabolism of linamarin for root protein synthesis, we observed a 2 fold increase in root total free amino acids in transgenic plants (Figure 5A) associated with an increase in total protein in transgenic cassava roots (Figure 6A).

Several approaches have been attempted to reduce the cyanogen content of cassava roots including inhibition of linamarin synthesis and accelerating cyanogensis and cyanide volatilization during root processing in transgenic plants [8], [20], [22]. Cassava foods are most toxic when short-cut approaches are used to remove cyanogens. The most abundant cyanogen in poorly processed cassava is typically acetone cyanohydrin, the substrate for HNL [11]. Virtually no free cyanide is ever detected in cassava foods since it is volatile [11]. The accumulation of acetone cyanohydrin in poorly processed cassava is the result of both insufficient spontaneous decomposition either due to the low pH or reduced temperatures or short processing time, and the lack of HNL activity in roots. When cassava roots were processed at low pH (<5) and temperatures (<25 C), conditions favorable for the accumulation of acetone cyanohydrin, we observed that HNL transgenic lines had a 80–90% reduction in acetone cyanohydrin levels (Figure 4A) relative to wild-type plants. In rubber plants, it is also suggested that reduction of cyanogenic glucosides can be achieved through blocking the transport of linustatin (linamarase-insensitive transport form) from the shoot apex [36], [37]. Till date, however, linustatin has not been detected in cassava [7]. Furthermore, since linamarin provides reduced nitrogen for root protein synthesis a strategy fro reducing cyanogen toxicity of roots based on blocking linustatin transport would likely result in less productive plants [21].

Overall, we have shown that targeted expression of HNL in roots leads to; 1) reduced steady-state linamarin levels consistent with the assimilation of the linamarin nitrile group into amino acids and protein via the β-cyanoalanine synthesis pathway, 2) an elevation of HNL protein levels, and 3) a substantial reduction in cyanogen levels in processed cassava roots (Figure 7). Since HNL is localized in the apoplast, it presumably is not subject to proteolytic turnover in intact tissues. Thus, targeting a root storage protein to the apoplast is proposed to be an effective strategy to hyper-accumulate proteins in cassava roots. In addition, HNL has a well-balanced amino acid composition for human nutrition (Figure S6). Since HNL is an unusually high-temperature stable enzyme, its expression in cassava roots is compatible with a variety of food processing technologies that accelerate cyanogen detoxification [9].

## Materials and Methods

### Plasmid construction and cassava transformation

Plasmid pBI121 carrying cassava Hydroxy Nitrile Lyase (HNL) cDNA [9] cloned between the tuber specific patatin promoter and nos terminator was amplified by adding *KpnI* and *PstI* sites to the primers (HNL-F: 5′ GAGACTGCAGTTGTAGTTAATGCGTATTAGTTTTAGC 3′ and HNL-R: 5′ TCTCGGTACCGATCTAGTAACATAGATGACACCGCG 3′). PCR amplified product was cloned at *KpnI* and *PstI* sites in pCambia2300. The arrangement of genes within the T-DNA region of pCambia 2300 vector from left border to right border is 2XCaMV35S: nptII: tNOS: patatin: *HNL*: tnos. The modified binary vector was mobilized into *Agrobacterium tumefaciens* strain LBA4404 (Life Technology, Grand Island, NY, USA) by electroporation and used to transform cassava cultivar TMS60444 through a friable embryogenic callus system [38].

### Real time-PCR analysis

Total RNA from leaves and roots of 12 Patatin:HNL transgenic lines, wild-type-60444 and 35S-HNL transgenic line [20] was isolated using the RNA-easy kit from Qiagen (Qiagen Inc., Valencia, CA, USA) according to the manufacturer's instructions. To remove contaminating genomic DNA, RNAs were treated with the DNAase I (Promega, Madison, WI, USA) according to the manufacturer's instructions. The concentrations of RNAs were assessed using a Nanodrop-2000C (Thermo-scientific, Wilmington, DE, USA) according to the manufacturer's instructions. The structural integrity of the RNAs was checked with non-denaturing agarose gel and ethidium bromide staining. DNase- treated RNA samples (0.5 µg) were reverse transcribed with an anchored oligo (dT) primer and 200 units superscript II reverse transcriptase (Invitrogen, Carlsbad, CA, USA) in a volume of 20 µl according to the manufacturer's instructions. Real-time quantitative RT-PCR was carried out using an ABI – Step One Plus (Applied Biosystems, Foster City, CA, USA) using PerfeCTa^TM^ SYBR® Green FastMix^ TM^ (ROX dye) (Quanta Biosciences, Gaithersburg, MD, USA) according to manufacturer's instructions. The cassava tubulin gene (Tub-F: 5′ GTGGAGGAACTGGTTCTGGA 3′ and Tub-R: 5′ TGCACTCATCTGCATTCTCC 3′) was used as reference gene/internal control and was amplified in parallel with the target HNL gene (HNL F: 5′ CAAACCAGCCCTTGAGAGAG 3′ and HNL-R: 5′ TTCCCCTTGAGGGAGTTTCT 3′) allowing gene expression normalization and providing quantification. Reactions were carried out using 50–100 ng/µl RNA in a final volume of 20 µl. All the primers were designed using the Primer Express software following the manufacturer's guidelines. (http://frodo.wi.mit.edu/cgi-bin/primer3/primer3_www.cgi). For each sample, reactions were set up in quadruplicates and two biological experiments were done to ensure the reproducibility of the results. The quantification of the relative transcript levels was performed using the comparative C_T_ (threshold cycle) method [39].

### HNL enzymatic activity

HNL enzymatic activity was performed according to Siritunga et al., 2004 [20]. Transformed and non-transformed cassava tissues (roots and leaves) from 5-month old plants (100 mg) were frozen in liquid nitrogen and ground in 0.5 mL of 0.05 M sodium phosphate buffer pH 5.0, 3 mM DTT, and 1% (w/v) polyvinyl pyrrolidine at 4°C. The cell wall material was pelleted by centrifugation at 13000 g for 15 min at 4°C. The supernatant was collected and centrifuged again to remove the cell debris. Supernatant protein concentrations were determined by CB-X™ Protein Assay (G-Biosciences, Maryland Heights, MO, USA) according to manufacturer's instructions. Hydroxynitrile lyase assays were performed in a final volume of 1 mL containing 50 mM sodium phosphate buffer pH 5.0, 20 µg total leaf protein, and 28 mM acetone cyanohydrin (Sigma, St. Louis, MO, USA). After 30 min incubation at 28°C in capped tubes, 10 µL of the reaction mixture was added to 100 µL glacial acetic acid. 400 uL of reagent A (50 mg of succinimide and 125 mg N-Chlorosuccinimide in 50 mL water) and 400 uL of reagent B (3 g barbituric acid and 15 mL pyridine in 35 mL water) was added to the reaction mixture and incubated for 5 minutes. Enzyme activity was measured colorimetrically by measuring the absorbance at 585 nm. KCN (10 µg/mL to 0.1 µg/mL) was used as a standard to obtain a linear curve and this equation was used to measure the amount of acetone cyanohydrin derived cyanide in cassava root materials.

### Extraction and quantification of protein

Cassava roots and leaves (10–15 mg) of 3, 5 and 7 month old transformed and non-transformed plants were homogenized in a mortar and pestle with protein extraction buffer (200 mM NaCl, 1 mM EDTA, 0.2% (v/v) Triton-X, 100 mM Tris-HCl (pH 7.8), 4% (v/v) 2-mercaptoethanol, supplemented with complete protease inhibitor cocktail (Roche, Basel, Switzerland). Tissues were extracted with 1 mL of extraction buffer with ceramic beads using a Fast Prep®-24 tissue and cell homogenizer (MP Biomedicals, Solon, OH, USA) at 5 m/s for 40 seconds. Samples were vortexed at 4°C for 10 min and centrifuged at 9000 rpm for 10 min at 4°C and supernatant was collected into a new tube. Extraction was repeated with another 1 mL extraction buffer. Supernatant protein concentrations were determined by CB-X™ Protein Assay (G-Biosciences, Maryland Heights, MO, USA) according to manufacturer's instructions.

### Quantification of total amino acids and free amino acids

Seven month old cassava transgenic and control lines (root tissues) were subjected for protein hydrolysis and for free amino acid analysis. For hydrolyzed proteins, samples were hydrolyzed for 24 h at 116°C in 6 N HCl containing 0.5% (v/v) phenol. Samples were dried and resuspended in 20 mM HCl, derivatized with the AccQ-tag reagent (Waters) and separated by ACQUITY UPLC® System (Waters, Milford, MA, USA) according to manufacturer's instructions. Samples were prepared for free amino acid analysis according to Hacham et al., 2002 [40] and analyzed on a ACQUITY UPLC® System (Waters, Milford, MA, USA) according to manufacturer's instructions. Triplicates were maintained for each biological sample.

### Western blot

Five month old-cassava transgenic and control lines were used for Western blot analyses. Total protein was extracted as explained earlier. 5–20 µg of soluble protein were resuspended in 40 µl of sample buffer (0.06 M Tris-HCl, pH 6.8, 10% (v/v) glycerol, 2% (w/v) SDS, 5% (v/v) 2-mercaptoethanol, 0.0025% (w/v) bromophenol blue) and heated at 95°C for 5 minutes. Samples were centrifuged for 30 seconds at 15,000 g to remove debris and sample was then separated by SDS-PAGE using 10% ready cast gels (Bio-Rad, Hercules, CA, USA) at 20 mA for 3 hrs. Proteins were electrophoretically transferred onto a PVDF membrane using a semi-dry transfer apparatus at 1.9–2.5 mA/cm^2^ of gel area for 60 minutes. The membrane was incubated for 1 hr in blocking solution (TBS: 20 mM Tris-HCl, pH 7.5, 500 mM NaCl, plus 0.5% (w/v) BSA). Membrane was incubated for 24 hr at 4°C with anti-HNL antibody diluted 1∶1000 in TTBS containing 0.5% (w/v) BSA (TBS containing 0.05% (v/v) Tween-20). Membrane was washed with TTBS three times (15 min. each) and secondary antibody (Anti-rabbit IgG antibody (Sigma, St. Louis, MO, USA) conjugated to horse-radish peroxidase) at 1∶10000 dilution was added at room temperature for 2 h. Membrane was washed with TTBS three times (15 min each) and detection was performed by chemiluminescence. Equal volumes of Solution A (100 mM glycine pH 10; 0.4 mM luminal and 8 mM iodophenol) and Solution B (0.12% (v/v) hydrogen Peroxide in water) were mixed and the membrane was incubated for 1 min. Excess liquid is drained and membrane was exposed to X-ray film [41].

### Measurement of free cyanide and acetone cyanohydrin in roots following maceration

Seven month-old transgenic and control plants were used for cyanide and acetone cyanohydrin assays. Root cortex tissues (1 gram) was extracted with 5 mL of 0.1 M sodium phosphate buffer pH 5.0 for 30 s by adding ceramic beads using Fast Prep®-24 tissue and cell homogenizer (MP Biomedicals, Solon, OH, USA) 5 m/s for 40 seconds and incubated at 30°C for 0–90 min in capped tubes. Starch was pelleted and removed by centrifugation at 7500 *g* for 2 min. The supernatant was immediately subjected to two assays. Liberated cyanide, a measure of acetone cyanohydrin decomposition, was measured by adding 0.5 mL of supernatant to 3.5 mL of 0.1 M sodium phosphate pH 5.0, followed by cyanide quantification using colorimetric method. Sample of the reaction mixture (100–500 µl) was added to 100 µl glacial acetic acid, 400 uL of reagent A (50 mg of succinimide and 125 mg N-chlorosuccinimide in 50 mL water) and 400 uL of reagent B (3 g barbituric acid and 15 mL pyridine in 35 mL water). Reaction mixture was incubated for 5 minutes and free cyanide was measured colorimetrically by measuring the absorbance at 585 nm. Total acetone cyanohydrin plus cyanide was determined by adding 0.1 mL of supernatant to 0.6 mL of 0.2 M NaOH and 3.3 mL of 0.1 mM sodium phosphate buffer pH 5.0, followed by cyanide quantification using the colorimetric method described earlier. The addition of NaOH converts all the acetone cyanohydrin into free cyanide. The amount of acetone cyanohydrin present in the HNL transformants was calculated as the difference between the two assays {(acetone cyanohydrin + cyanide assay) – (cyanide assay)}.

#### Measurement of linamarin content

Seven month old transgenic and control cassava lines were used to measure linamarin content. Both leaves and root tissues (10–25 mg dry weight) were used for solvent extraction. Acetonitrile (250 µl) was added to the dry lyophilized powder and extracted twice for 30 min by shaking. Samples were centrifuged and supernatant was transferred to a new tube. Supernatant was dried using a CentriVap DNA Concentrator (Labconco, Kansas city, KS, USA) and dissolved with water, chloroform and 10 µL of phenyl β-glucopyranoside (PGP). Samples were mixed, centrifuged and the upper phase was again dried using a CentriVap DNA Concentrator (Labconco, Kansas city, KS, USA). Samples were redissolved with 50 µL of acetonitrile and derivatized with 50 µL of MSTFA+1% TMCS (Pierce, Rockford, IL, USA) and 10 µL of pyridine at 65°C for 30 min on a dry heating block. GC-MS analysis was performed using an Agilent 5975C Series instrument (Agilent Technologies, Santa Clara, CA, USA). A 30 meter long, 0.25 micron film thickness ZB-5MSi Zebron® Guardian with integrated guard capillary gas chromatography column (#7HG-G018-11-GGA; Agilent Technologies, Santa Clara, CA, USA) at an injection temperature of 250°C was used for separation. The GC-MS was operated under a pressure control mode using pressures that gave flow rates near 1 mL/minute. The GC oven temperature program was: 50°C for one minute after injection, ramp at 30°C/minute to 185°C, ramp at 6°C/minute to 230°C (linamarin elution), ramp at 12°C/minute to 280°C (internal standard elution), and 3 minutes at 290°C to clean the column. Standards are prepared with different concentrations of phenyl β-glucopyranoside (PGP) and linamarin (Sigma). The peak areas of linamarin and PGP was plotted to obtain a linear curve and this equation was used to measure the amount of linamarin in transgenic and control lines.

## Supporting Information

Figure S1 MEGA4 (Molecular Evolutionary Genomic Analysis) multiple amino acid sequence alignments of cassava HNL and rubber HNL. The HNL proteins share 77% sequence identity.
(DOC)

Figure S2 (A). Schematic graph depicting the copy numbers of the HNL transgenic lines analyzed by dot blots. (B). Sample of PCR showing the presence of HNL and patatin gene in the transgenic lines. Controls included water (Negative control), WT-60444 and plasmid (Positive control).
(DOC)

Figure S3 GC-MS quantification of leaf linamarin content in wild-type and transgenic plants. Samples were normalized with internal standard phenyl β-glucopyranoside (PGP). Linamarin content is expressed as µmoles per gram dry weight. Error bars indicate SE of the mean of four biological replicates.
(DOC)

Figure S4 Total amino acids composition of roots of transgenic cassava plants and wild type controls. Error bars represent SE for two biological replicates. Each amino acid is expressed using three letter abbreviation.
(DOC)

Figure S5 Free amino acids composition of roots of transgenic cassava plants and wild type controls. Error bars represent SE for two biological replicates. Each amino acid is expressed using three letter abbreviation.
(DOC)

Figure S6 Predicted amino acid composition of HNL protein using ProtParam tool (ExPasy proteomics Server). (A). Shows the classification of HNL protein into essential and non-essential amino acids. (B). shows the individual% of amino acid composition of essential amino acids
(DOC)

Methods S1 Supplemental Methods.
(DOC)
