# Supplementary figures and images for: Overexpression of Hydroxynitrile Lyase in Cassava Roots Elevates Protein and Free Amino Acids while Reducing Residual Cyanogen Levels
Source: PLoS One. 2011 Jul 25;6(7):e21996. doi: 10.1371/journal.pone.0021996 (PMC3143114; doi:10.1371/journal.pone.0021996)

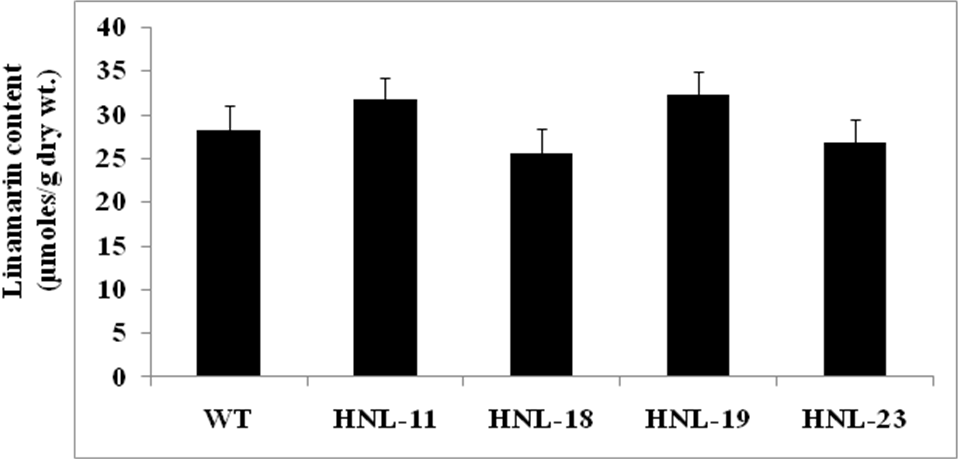

Supplement: Figure S3 — GC-MS quantification of leaf linamarin content in wild-type and transgenic plants. Samples were normalized with internal standard phenyl β-glucopyranoside (PGP). Linamarin content is expressed as µmoles per gram dry weight. Error bars indicate SE of the mean of four biological replicates. (DOC) [file pone.0021996.s003.doc]
