# Supplementary material for: Overexpression of Hydroxynitrile Lyase in Cassava Roots Elevates Protein and Free Amino Acids while Reducing Residual Cyanogen Levels
Source: PLoS One. 2011 Jul 25;6(7):e21996. doi: 10.1371/journal.pone.0021996 (PMC3143114; doi:10.1371/journal.pone.0021996)
Supplement: Methods S1 — Supplemental Methods. (DOC) [file pone.0021996.s007.doc]

**Supplemental Methods**

**PCR Analysis**

To identify the presence of the transgene, PCR was carried out using the genomic DNA isolated from 45d old *invitro* plants. Total genomic DNA from leaf tissues was isolated using the DNeasy Plant Mini Kit from Qiagen (Qiagen Inc., Valencia, CA, USA) according to the manufacturer’s instructions. DNA from the untransformed plants was used as a negative control and plasmid DNA of pCambia 2300 carrying Patatin-HNL-NOS were used as positive control. PCR analysis were performed employing gene specific HNL primers (HNL F: 5’ AAGCTCAAACCAGCCCTTG 3’ and HNL R: 5’ AATTTGCCAGCGTTGAAAGT 3’) and patatin primers (Pat-F: CGTCTCACAAAATTTTTAGTGACG 3’ and Pat-R: 5’ TGATGTTTATTATCTCACTCACTTTGC 3’). The reaction mixture containing template, primers, buffer, dNTPs and Taq DNA polymerase was subjected to initial denaturation (94º C) for 4 min, followed by repeated denaturation (94º C) for 30s, annealing (53º C) for 30s, and elongation (72º C) for 1 min for a total of 35 cycles. Final elongation step was carried out at (72º C) for 10 min. Amplified PCR products were analysed by gel electrophoresis on 1.0% agarose gel.

**Dot Blot Analysis**

To identify the copy number of the transgenic plants, genomic DNA (100 ng) was used as template for dot blot. DNA was denatured with equal volume of 0.4 M NaOH by boiling for 5-10 min. and tubes were placed immediately onto ice for 5 min. 100µl of 2XSSC was added to each DNA sample and blotted to nylon membrane (Hybond N+) using a Bio-Dot ® Micro filtration apparatus (Bio-Rad, Hercules, CA) according to the manufacturer’s instructions. The membranes were washed with 2X SSC (300 mM NaCl, 30 mM sodium citrate). DNA was crosslinked to membrane using an UV Stratalinker (auto-crosslink setting) and stored at room temperature. Three replicates were carried out for each DNA sample. Cassava TMS 60444 lines carrying 35S with 0, 1, 2 and 3 copies (Gift from Mohammed Abhary, DDPSC) were used as controls. 2X35S probe was amplified using 35S specific primers (35S-F: 5’ CACATCAATCCACTTGCTTTGAAG 3’ and 35S-R: 5’ CATGGTGGAGCACGACACT 3’). Probe synthesis, hybridization, washing were done using the DIG High prime DNA labeling and Detection Starter Kit II (Roche Applied Science, Indianapolis, IN, USA) according to manufacturer’s instructions. The final detection was done by chemiluminescence (1:150) dilution of CDP-Star Reagent (Roche Diagnostics) followed by exposure to X-ray films. The films were scanned using Epscon Scanner and Spot finding, quantification and background subtraction was done using Image J (<http://rsbweb.nih.gov/ij/index.html>; Image processing and analysis in Java). Standard curve equation was obtained between the copy numbers and spot intensities using the standard control plants carrying 0, 1, 2 and 3 copies. This equation was used to calculate the copy numbers in the transgenic lines.
