# Supplementary material for: Overexpression of Hydroxynitrile Lyase in Cassava Roots Elevates Protein and Free Amino Acids while Reducing Residual Cyanogen Levels
Source: PLoS One. 2011 Jul 25;6(7):e21996. doi: 10.1371/journal.pone.0021996 (PMC3143114; doi:10.1371/journal.pone.0021996)
Supplement: Supplementary file 3 [file pone.0021996.s001.doc]

```
MeHNL : MVTAHFVLIHTICHGAWIWHKLKPALERAGHKVTALDMAASGIDPRQIEQINSFDEYSEP :  60
HbHNL : MAFAHFVLIHTICHGAWIWHKLKPLLEALGHKVTALDLAASGVDPRQIEEIGSFDEYSEP :  60

MeHNL : LLTFLEKLPQGEKVIIVGESCAGLNIAIAADRYVDKIAAGVFHNSLLPDTVHSPSYTVEK : 120
HbHNL : LLTFLEALPPGEKVILVGESCGGLNIAIAADKYCEKIAAAVFHNSVLPDTEHCPSYVVDK : 120

MeHNL : LLESLPDWRDTEYFTFTNITGETITTMKLGFVLLRENLFTKCTDGEYELAKMVMRKGSLF : 180
HbHNL : LMEVFPDWKDTTYFTYT-KDGKEITGLKLGFTLLRENLYTLCGPEEYELAKMLTRKGSLF : 179

MeHNL : QNVLAQRPKFTEKGYGSIKKVYIWTDQDKVFLPDFQRWQIANYKPDKAYQVQGGDHKLQL : 240
HbHNL : QNILAKRPFFTKEGYGSIKKIYVWTDQDEIFLPEFQLWQIENYKPDKVYKVEGGDHKLQL : 239

MeHNL : TKTEEVAHILQEVADAYA : 258
HbHNL : TKTKEIAEILQEVADTYN : 257
```
