# Supplementary material for: Overexpression of Hydroxynitrile Lyase in Cassava Roots Elevates Protein and Free Amino Acids while Reducing Residual Cyanogen Levels
Source: PLoS One. 2011 Jul 25;6(7):e21996. doi: 10.1371/journal.pone.0021996 (PMC3143114; doi:10.1371/journal.pone.0021996)
Supplement: Supplementary file 4 [file pone.0021996.s002.doc]

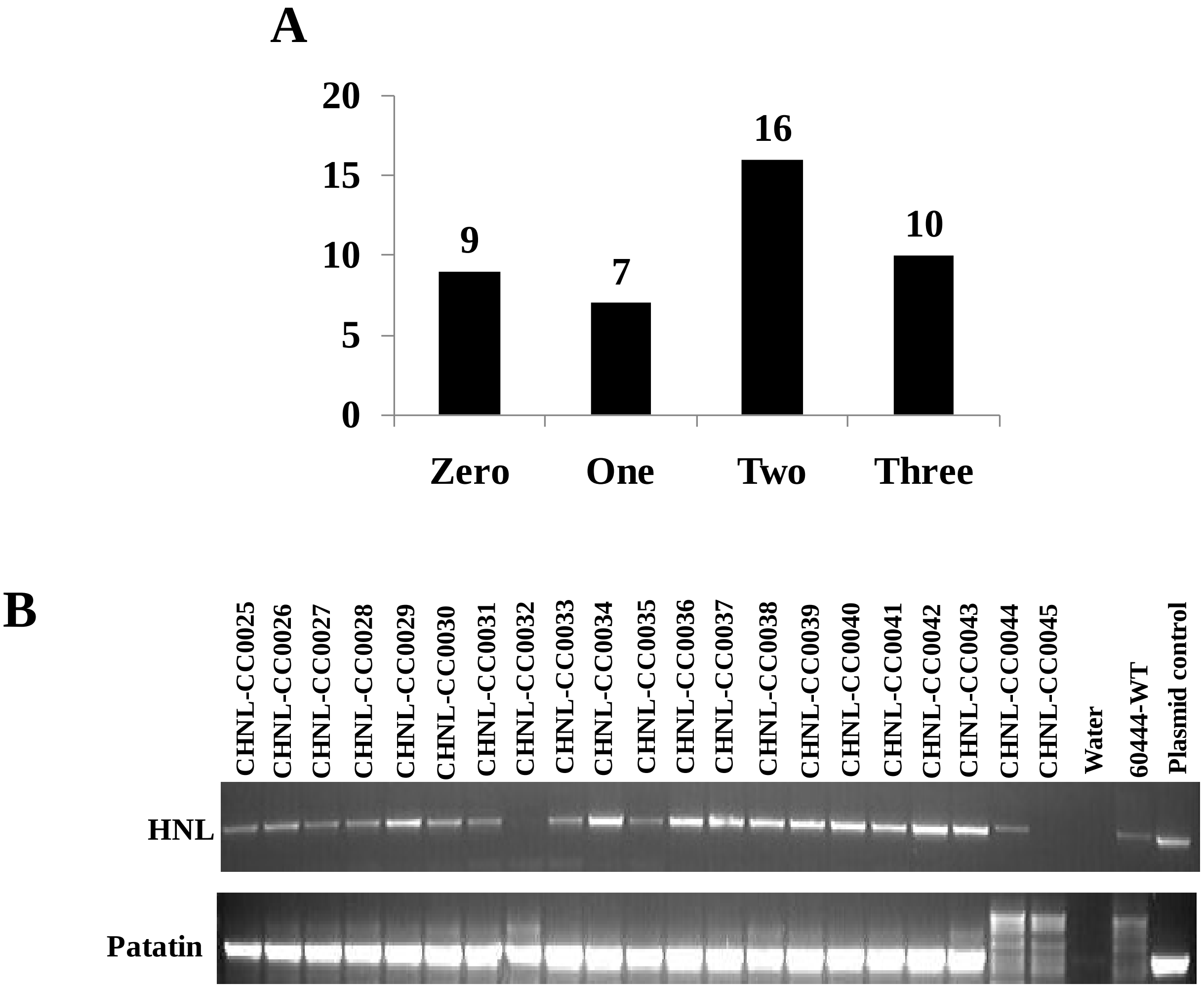
A
20
15
10
5
0
9
7
16
10
Zero
One
Two
Three
B
CHNL-CC0025
CHNL-CC0026
CHNL-CC0027
CHNL-CC0028
CHNL-CC0029
CHNL-CC0030
CHNL-CC0031
CHNL-CC0032
CHNL-CC0033
CHNL-CC0034
CHNL-CC0035
CHNL-CC0036
CHNL-CC0037
CHNL-CC0038
CHNL-CC0039
CHNL-CC0040
CHNL-CC0041
CHNL-CC0042
CHNL-CC0043
CHNL-CC0044
CHNL-CC0045
Water
60444-WT
Plasmid control
HNL
Patatin
