# Supplementary material for: Overexpression of Hydroxynitrile Lyase in Cassava Roots Elevates Protein and Free Amino Acids while Reducing Residual Cyanogen Levels
Source: PLoS One. 2011 Jul 25;6(7):e21996. doi: 10.1371/journal.pone.0021996 (PMC3143114; doi:10.1371/journal.pone.0021996)
Supplement: Supplementary file 5 [file pone.0021996.s004.doc]

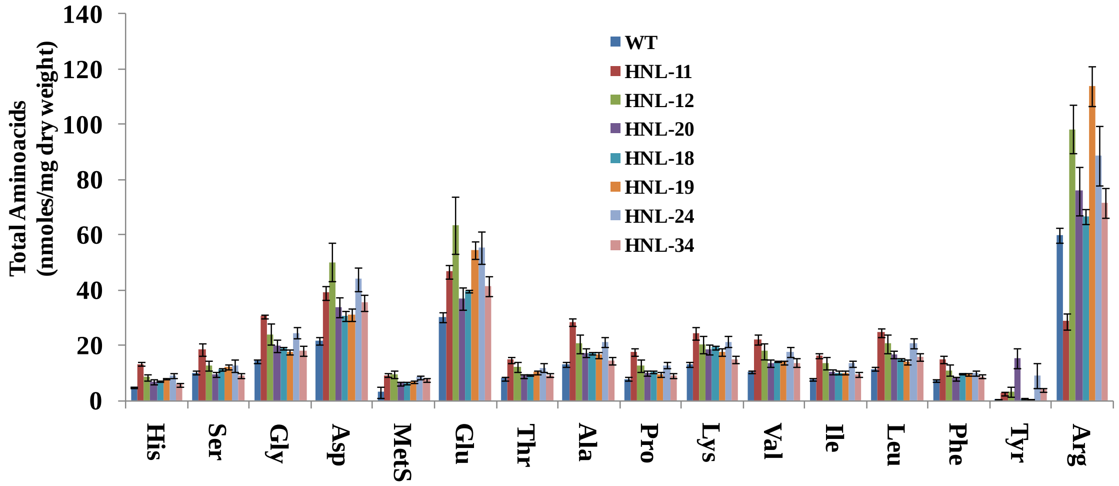

Total Aminoacids (nmoles/mg dry weight)
140
120
100
80
60
40
20
0
WT
HNL-11
HNL-12
HNL-20
HNL-18
HNL-19
HNL-24
HNL-34
His
Ser
Gly
Asp
MetS
Glu
Thr
Ala
Pro
Lys
Val
Ile
Leu
Phe
Tyr
Arg
