# Supplementary material for: Overexpression of Hydroxynitrile Lyase in Cassava Roots Elevates Protein and Free Amino Acids while Reducing Residual Cyanogen Levels
Source: PLoS One. 2011 Jul 25;6(7):e21996. doi: 10.1371/journal.pone.0021996 (PMC3143114; doi:10.1371/journal.pone.0021996)
Supplement: Supplementary file 6 [file pone.0021996.s005.doc]

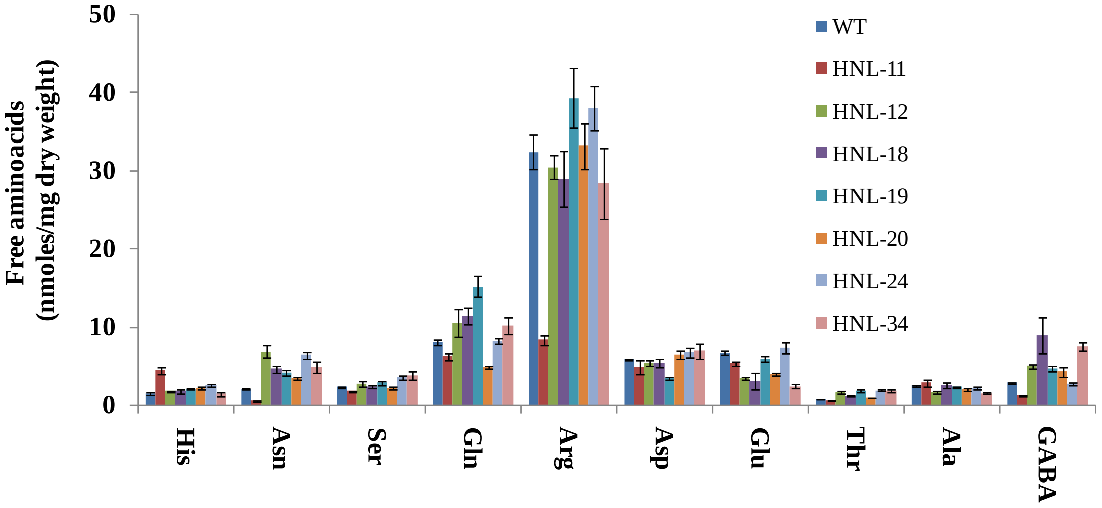
Free aminoacids
(nmoles/mg dry weight)
50
40
30
20
10
0
WT
HNL-11
HNL-12
HNL-18
HNL-19
HNL-20
HNL-24
HNL-34
His
Asn
Ser
Gln
Arg
Asp
Glu
Thr
Ala
GABA

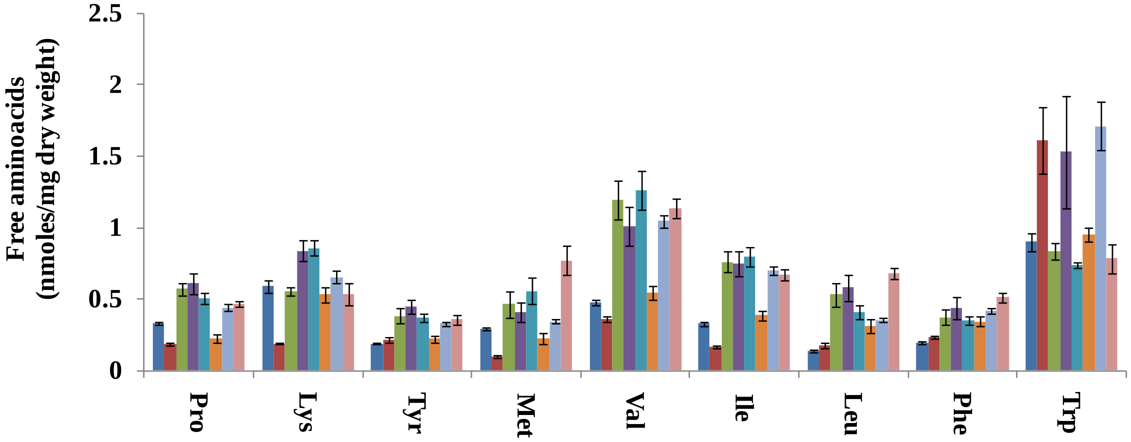
Free aminoacids
(nmoles/mg dry weight)
2.5
2
1.5
1
0.5
0
Pro
Lys
Tyr
Met
Val
Ile
Leu
Phe
Trp
