# Supplementary material for: Overexpression of Hydroxynitrile Lyase in Cassava Roots Elevates Protein and Free Amino Acids while Reducing Residual Cyanogen Levels
Source: PLoS One. 2011 Jul 25;6(7):e21996. doi: 10.1371/journal.pone.0021996 (PMC3143114; doi:10.1371/journal.pone.0021996)
Supplement: Supplementary file 7 [file pone.0021996.s006.doc]

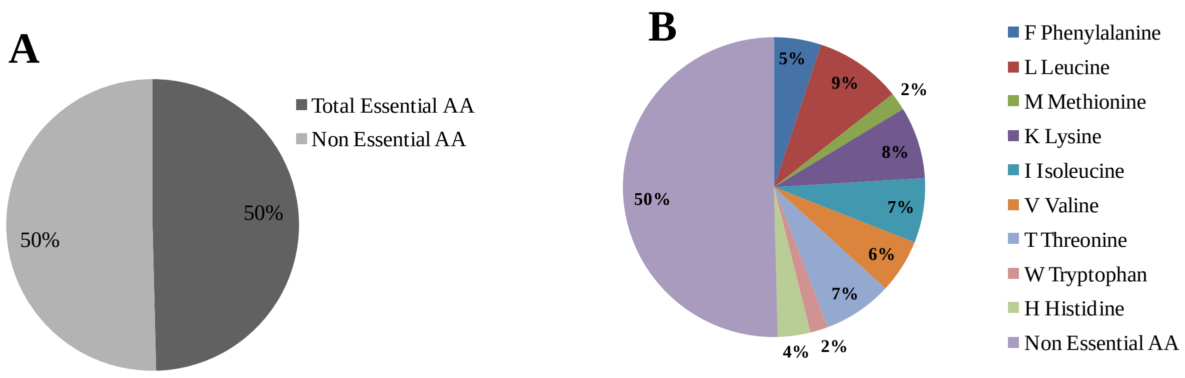
A
50%
50%
Total Essential AA
Non Essential AA
B
5%
9%
2%
8%
7%
6%
7%
2%
4%
50%
F Phenylalanine
L Leucine
M Methionine
K Lysine
I Isoleucine
V Valine
T Threonine
W Tryptophan
H Histidine
Non Essential AA
